# Pain in Hemodialysis Patients: Prevalence, Intensity, Location, and Functional Interference in Daily Activities

**DOI:** 10.3390/healthcare9101375

**Published:** 2021-10-14

**Authors:** Patrícia Roberta dos Santos, Carolina Rodrigues Mendonça, Matias Noll, Cezimar Correia Borges, Polissandro Mortoza Alves, Naiara Toledo Dias, Amanda Maria de Sousa Romeiro, Maria Alves Barbosa, Celmo Celeno Porto

**Affiliations:** 1 Programa de Pós-Graduação em Ciências da Saúde, Faculdade de Medicina, Universidade Federal de Goiás, Goiânia 74605-010, Goiás, Brazil; borgescezimar@gmail.com (C.C.B.); uegmortoza@gmail.com (P.M.A.); maria.malves@gmail.com (M.A.B.); celeno@cardiol.br (C.C.P.); 2 Instituto Federal Goiano, Ceres 76300-000, Goiás, Brazil; matias.noll@ifgoiano.edu.br; 3 Department of Sports Science and Clinical Biomechanics, University of Southern Denmark, 5230 Odense, Denmark; 4 Curso de Educação Física, Universidade Estadual de Goiás, Itumbiara 75533-000, Goiás, Brazil; 5 Programa de Pós-Graduação em Ciências da Saúde, Universidade Federal de Uberlândia, Uberlândia 38408-100, Minas Gerais, Brazil; naiaratdias@gmail.com; 6 Curso de Enfermagem, Universidade Estadual de Goiás, Itumbiara 75533-000, Goiás, Brazil; romeiroamanda@hotmail.com

**Keywords:** renal insufficiency, hemodialysis, chronic, renal dialysis, pain, activities of daily living, adults, epidemiology, prevalence

## Abstract

Although pain is a frequent complaint of patients with chronic kidney disease who undergo hemodialysis, few studies have assessed the functional interference of pain in activities of daily living (ADLs). Hence, the aim of this study was to evaluate the prevalence, location, intensity, and functional interference of pain in ADLs of chronic kidney disease patients undergoing hemodialysis and to estimate the association of specific pain sites with severe functional interference by pain in ADLs. This cross-sectional study included patients with chronic kidney disease undergoing hemodialysis. The prevalence, intensity, and functional interference of pain in ADLs were assessed using the brief pain inventory. Poisson regression was used to calculate the prevalence ratio. A total of 65 patients participated in the study. The overall prevalence of pain was 89.23%; the prevalence of headache was 18.46% and that of pain in the trunk was 55.38%, upper limbs was 35.38%, and lower limbs was 60.00%. The prevalence of moderate and severe pain at the time of hemodialysis was 13.85% and 21.54%, respectively. A high prevalence of severe functional interference of pain in general activity (61.54%), mobility (56.92%), and disposition (55.38%) was observed. Pain is a frequent complaint in patients undergoing hemodialysis, mainly musculoskeletal and intradialytic, and it interferes with ADLs and incapacitates the patient. Pain was highly prevalent in the upper and lower limbs and the trunk. Furthermore, a higher prevalence of severe pain at the time of hemodialysis and functional interference of pain, mainly in general activity, mobility, and disposition, were observed.

## 1. Introduction

Chronic kidney disease (CKD) is a public health problem [1] with a global prevalence of 9.1% (697.5 million cases) and is the 12th leading cause of death worldwide [2]. In Brazil, the number of patients undergoing dialysis increased from 42,695 in 2000 to 91,314 in 2011 [3], reaching 133,464 in 2018 [4]. In addition, Brazil is the third country in the world in the number of patients requiring dialysis in the long term [4]. In CKD, changes in glomerular filtration rates, whether or not associated with parenchymal changes, for more than 3 months [5,6] require treatment via renal replacement therapy by peritoneal dialysis or hemodialysis (HD) for maintaining homeostasis and increasing patient survival [7]. However, in several countries, HD is an experience that influences the quality of life and can be painful [8].

Patients with end-stage renal disease may present with pain related to renal bone disease (osteitis fibrosa cystica, amyloidosis, and osteomalacia), osteoarthritis, calcific uremic arteriolopathy, and peripheral neuropathy. In addition, comorbidities such as ischemic peripheral artery disease, diabetic neuropathy, osteopenia/osteoporosis (due to long-standing hypertension, diabetes, or old age) result in different types of pain. Furthermore, primary kidney disease as well as hemodialysis or peritoneal dialysis are important causes of pain [9]. Pain in patients with CKD varies in location and intensity. Pain in these patients could manifest as myalgia, cramps, headache, musculoskeletal pain, neuropathic pain, and/or chest pain [10,11]. In addition, bone pain and progressive loss of muscle mass can also affect the quality of life of these patients [12,13,14].

Although pain is a frequent complaint of CKD patients undergoing HD, few studies in the literature have analyzed the presence of pain, especially how it interferes with the activities of daily living (ADLs) [11,15,16]. Thus, in this study, we aimed to assess the prevalence, location, intensity, and functional interference of pain in the ADLs of CKD patients undergoing HD and to analyze the association of specific pain sites with severe functional interference of pain in ADLs. Such studies can improve our understanding of pain in patients undergoing HD, enabling the institution of effective measures for its prevention and treatment.

## 2. Materials and Methods

### 2.1. Study Design

This cross-sectional study was performed with data of patients at a Nephrology Clinic in the Midwest region of Brazil, which serves patients through the Sistema Único de Saúde or SUS (the Brazilian public health system). A total of 85 patients were interviewed. Data were collected from June to September 2018. This research with human subjects was conducted in compliance with the Helsinki Declaration. This research was approved in October 2016 by the Ethics Committee of the Hospital das Clínicas of the Federal University of Goiás (Goiânia, Goiás, Brazil), CAAE 59756416.1.0000.5083; with the protocol number 1,782,067.

### 2.2. Inclusion and Exclusion Criteria

Adult patients aged over 18 years, with end-stage renal disease, undergoing HD for 6 months or more, and with no other known disease were included. Patients with any type of disability that could interfere with data collection, patients with cancer, HIV, and/or neurological diseases, and patients who did not undergo treatment at the service during data collection were excluded.

### 2.3. Measures

Sociodemographic, lifestyle, economic, family, and social profile data were collected using a standardized questionnaire that was prepared by the authors and was previously tested. Sociodemographic and lifestyle variables were age, sex, marital status, education, and physical activities (yes/no). In the assessment of economic profile, whether the patient’s spouse worked outside the home, whether he/she had his/her own home, whether he/she was paid for his/her work, and the salary range were enquired [17]. While evaluating the family and social profile, the number of family members living at the residence, number of children, and religious practice and if it had any social benefits were determined.

The brief pain inventory (BPI) was used to assess the prevalence, intensity, location, and functional interference of pain in the ADLs of CKD patients undergoing HD. The BPI is an instrument developed by Daut et al. at the University of Wisconsin in 1983 to assess pain and its impact on the daily activities of cancer patients [18]. In 2004, it was validated for patients with chronic non-cancer pain [19,20]. In 2009, it was translated and validated in Portuguese (Brazil) by Ferreira-Valente et al. [21], and in 2017, the BPI was validated for patients with CKD [22].

The BPI assesses the location of pain (head, trunk, and upper and lower limbs), pain during HD, pain intensity in the past week, and the functional interference of pain in ADLs. The pain location (head, trunk, and upper and lower limbs) was determined via a diagram representing the human body, and the pain intensity was measured using a numerical rating scale from 0 to 10. Pain intensity in patients with HD was classified as no pain (score = 0), mild pain (score ≤ 3), moderate pain (scores between 4 and 6), and severe pain (score ≥ 7) [23].

Functional interference of pain was assessed on a scale of 0–10, where 0 implied “it did not interfere” and 10 implied “it completely interfered”. Functional interference was scored according to the scores for seven BPI questions, comprising the domains of physical interference [general activity, disposition, ability to walk (mobility), and work] and affective interference (relationships with other people, mood, and joy of living) [18]. Patients were allocated to the following groups according to their scores: no interference (0), little interference (<2), moderate interference (3–5), and severe interference (≥6), as described previously [24].

The seven items that assessed the functional interference of pain in the patients’ ADLs were considered independent variables. In addition, four outcomes were considered: headache (yes/no) or trunk (yes/no), upper limb (yes/no), or lower limb (yes/no) pain. All data were collected by the main researcher (PRS), during the HD of patients with CKD. Responses to the questionnaire were recorded in 20–35 min, and no difficulty was observed.

### 2.4. Statistical Analysis

The database was built using the EPI DATA^®^ version 3.1 program. The statistical package Stata version 16.0 (Stata Corp LP, College Station, TX, USA) was used for the analyses. Statistical significance was established at *p* < 0.05. Descriptive variables are presented in absolute numbers (*n*) and relative frequencies (%), with means and standard deviations. The Chi-square test (χ^2^) or Fisher’s exact test was used in the bivariate analysis of functional interference of pain in ADLs. Poisson regression was used to calculate the prevalence ratio (PR) and 95% confidence interval (CI) for the variable functional interference of pain in ADLs. Variables with *p* < 0.20 in the bivariate analysis were included in the multiple hierarchical Poisson regression analyses, with robust variance based on a hierarchical model [25]. Independent variables in this analysis included functional interference of pain in general activity, ability to walk, and relationships with other people. In the multivariate analysis, variables without statistical power were excluded (*n* < 10 in all strata) [26].

## 3. Results

Of the 85 interviewed patients, 20 were excluded (12 who did not consent to participate and eight who had difficulties in understanding and answering the questionnaires). Thus, 65 patients with CKD undergoing HD participated in this research, of which 33 (50.8%) were men and 32 (49.2%) were women. The participants were aged between 24 and 85 years with a mean age of 55 ± 1.83 years. The mean time for the patients were undergoing HD was 54.51 ± 6.04 months, and 26.2% (*n* = 17) patients underwent treatment for 6–24 months, 29.2% (*n* = 19) for 25–48 months, 21.5% (*n* = 14) for 49–72 months, and 23.1% (*n* = 15) for >73 months. In our sample, all patients had nephropathy, 80.0% (*n* = 52) had hypertension, 60.0% (*n* = 39) were diabetic, 4.6% (*n* = 3) had polycystic kidney disease, 13.8% (*n* = 9) had nephrosclerosis, and 56.9% (*n* = 37) had a musculoskeletal disease. Sixty-two patients (95.4%) received family support for HD treatment. The demographic and economic profiles are presented in Table 1, and the social profile is presented in Table 2.

### Pain Data

Of the investigated patients, 89.2% (*n* = 58) patients experienced some type of pain. The prevalence of headache was 18.5% (*n* = 12), that of trunk pain was 55.4% (*n* = 36), that of pain in the upper limbs was 35.4% (*n* = 23), and that of pain in the lower limbs was 60% (*n* = 39). In the past week, 73.8% (*n* = 48) patients experienced pain, whereas 26.5% (*n* = 17) had no pain. Of the 48 patients with pain, 38.5% (*n* = 25) patients had mild pain, 29.2% (*n* = 19) had moderate pain, and 6.2% (*n* = 4) had severe pain. The average maximum pain intensity in the past week was 6.11 ± 0.42 on the numerical rating scale, and the average minimum pain intensity was 2.77 ± 0.30. Pain intensity at the time of HD was mild in 6.2% (*n* = 4), moderate in 13.8% (*n* = 9), and severe in 21.5% (*n* = 14) patients. Most importantly, of the total number of patients, 26.2% (*n* = 17) had severe pain (visual analog scale—score 8–10), with longer dialysis time associated with higher pain prevalence. The presence of pre-existing musculoskeletal disease was associated with the presence of pain (*p* = 0.016). These patients continuously used analgesics prescribed by a physician and did not use opioids. Pain at the time of dialysis was present in 41.5% (*n* = 27) of patients.

**Figure 1:**
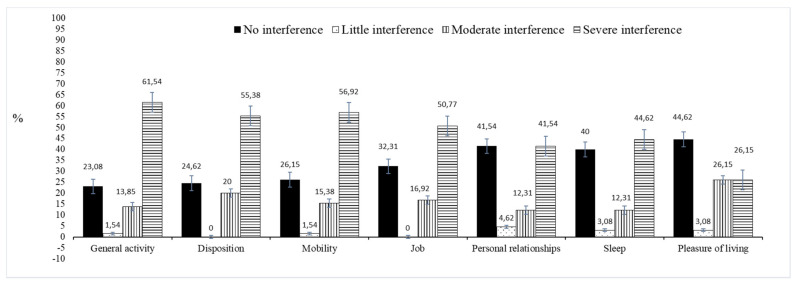
Prevalence of functional interference of pain in activities of daily living of patients with chronic kidney disease undergoing hemodialysis (*n* = 65).

Of the total number of patients, 24.6% (*n* = 16) could not perform ADLs independently. Functional interference of pain in the ADLs of patients with CKD undergoing HD was assessed using seven BPI variables, as shown in Figure 1. The prevalence of severe functional interference was high, primarily in the domains of physical interference in general activity (61.5%), ability to walk (56.9%), disposition (55.4%), and work (50.8%).

Poisson regression analysis revealed a significant association of severe functional interference of pain in the ability to walk with pain in the upper limb (*p* = 0.003). The prevalence and Poisson regression analysis for the association of pain sites with severe functional interference of pain in ADLs are shown in Table 3. Multiple regression analysis revealed that severe functional interference was not associated with general activity (PR 1.21, 95% CI 0.74–2.00, *p* = 0.446), ability to walk (PR 1.16, 95% CI 0.74–1.81, *p* = 0.521), and relationships with other people (PR 1.18, 95% CI 0.42–0.79, *p* = 0.419).

## 4. Discussion

This research focused on investigating the prevalence, location, intensity, and functional interference of pain in ADLs of patients with CKD undergoing HD. Furthermore, to the best of our knowledge, this is the first study to investigate the association of pain sites with severe functional interference in ADLs. The results of this study indicate that patients with CKD show a high prevalence of pain, especially in the upper and lower limbs and the trunk. The prevalence of pain of severe intensity at the time of HD and severe functional interference of pain, primarily in general activity, mobility, and disposition, was high. In addition, a significant association was observed between pain in the upper limb and its functional interference on the ability to walk.

Our findings of high prevalence of pain are consistent with other studies that have evaluated patients with CKD undergoing HD [11,12,27,28,29,30,31]. These studies have reported a prevalence of pain between 38% [29] and 95.6% [31]. In our study, the prevalence of headache was 18.46% and that of pain in the trunk was 55.38%, that of the upper limbs was 35.38%, and that of the lower limbs was 60.00%. Other studies have reported a higher prevalence of headache, with values of 32% [11], 53.6% [28], and 76.1% [32]. A study has reported a prevalence of 7% for pain in the upper limbs and 47% for pain in the lower limbs [33]. Other studies have found similar results to those of this study for the prevalence of trunk pain in patients with CKD [11,27]. Hence, pain reported by patients undergoing HD cannot be neglected.

In this study, a higher prevalence of severe pain was reported at the time of HD. This result is in line with other studies, as indicated by a systematic review [34]. In addition, a study conducted in 2009 showed that pain associated with the procedure and musculoskeletal pain were prevalent during and after the HD session [27]. It is known that the intensity of this pain can cause disability, affect the quality of life, and lead to exclusion from the job market [13].

A high prevalence of severe functional interference of pain was reported, primarily in general activity, mobility, and disposition. Other studies performed in Spain, Brazil, Switzerland, Argentina, and China have also investigated the interference of pain in ADLs [11,15,16,33,35]. These studies indicated the interference of pain in mood [11,35], usual work [35], social relationships [27], ability to walk, sleep, and work, and personal relationships [15].

Chronic and acute pain are common in patients undergoing HD, especially during puncture. Bone and abdominal pain may appear with high intensity and discomfort that interfere with sleep quality and ADLs [15,29,36,37]. Furthermore, the ability to walk was mostly affected, whereas whether the effect was light or moderate had divided opinions. Silva et al. [34] in 2013 reported that 57.5% of HD patients had chronic pain and 78.8% had intradialytic pain and that chronic pain interfered with mood, as well as with the ability to walk as shown by Dantas and Martins [15].

A significant association was observed between the functional interference of pain in the ability to walk and pain in the upper limb. We hypothesized that pain in patients undergoing HD could be limiting and disabling, ultimately affecting patient mobility. A recent systematic review suggests that acute and chronic pain is a prevalent complaint in adults and elderly people on HD and that the higher frequency of moderate and severe pain in different parts of the body interfered with daily activities [38]. These findings reinforce the fact that pain in patients undergoing HD must be considered and treated.

Pain management in patients with end-stage renal disease is a complex and challenging task, and effective pain and symptom control improves quality of life. Pain history assessment is the initial step in pain management, followed by the involvement of palliative care, patient and family counseling, discussion of treatment options, and correction of reversible causes. The first line of treatment should be conservative management, with exercises, physical therapy, acupuncture, meditation, music therapy, and cognitive behavioral therapy. If pain control is not optimal, replacement/addition of opioid analgesics is recommended. However, complex pain syndrome requires an analgesic regimen composed of polypharmacy with opioids, non-opioids, and adjuvant medications, which must be individualized to the patient to obtain adequate pain control [39].

Family support based on social care for the patient, considering their individual needs and coping possibilities, can help health teams with pain assessment and management in the family environment, helping to control and improve this symptom in patients undergoing HD [40]. With regard to institutional care, adequate assessment of the presence of pain and identification of the cause of pain in HD patients are important to design a care plan. This treatment plan can be pharmacological and non-pharmacological with the objective of reducing pain symptoms [41].

The strengths of this study are the inclusion of patients undergoing HD and assessment of severe functional interference of pain in ADLs, which is a novelty of this study. However, it also has some limitations that must be considered. First, as the study design was cross-sectional, a temporal relationship and inference of causality between variables could not be established. Second, the sample size was small, but representative, considering that they are patients undergoing HD. Third, the adoption of convenience sampling restricted the applicability of the survey results to the population. Fourth, data collection was carried out at a single HD service center in Brazil, which may limit the applicability of the findings to different HD centers, especially those that treat patients with high comorbidities. Despite these limitations, the results are clinically important, mainly because the high prevalence of pain and the non-use of opioids found in this study reveal the possible failure in adequate pain assessment of patients with CKD and, consequently, the possibility of inappropriate and ineffective prescription of medications. Further research, with a larger sample size, is needed to validate our findings. Furthermore, it is important that future studies investigate the causes of pain in patients with end-stage renal disease, influence of musculoskeletal diseases, amyloidosis, diabetic neuropathy, and physical activity, and use of medications. We encourage further studies on this topic in large HD centers.

We highlight the importance of adequate pain assessment in HD patients, as well as the inclusion of professionals who specialize in pain management in the multidisciplinary team. The adoption of pharmacological or non-pharmacological therapeutic approaches, such as the use of opioids and physiotherapy, can improve the symptoms of pain in HD patients and, consequently, have significant improvements in the quality of life of these patients.

## 5. Conclusions

This study showed that CKD patients undergoing HD have a high prevalence of pain, mainly musculoskeletal and intradialytic pain. A high prevalence of pain was also observed in the upper and lower limbs and the trunk. Furthermore, a higher prevalence of severe pain at the time of HD and functional interference of pain, mainly in general activity, mobility, and disposition, were observed. In addition, severe functional interference of pain in the ability to walk was associated with pain in the upper limb. These results indicate that pain in patients undergoing HD is limiting and disabling.

## Figures and Tables

**Table 1:** Characterization of the demographic and economic profile (*n* = 65).

Characterization	*n*	%
**Demographic profile**		
Age		
24 to 59 years	40	61.5
60 to 85 years	25	38.5
Sex		
Female	32	49.2
Male	33	50.8
Marital status		
Married	20	30.8
Companion	4	6.2
Divorced	14	21.5
Single	16	24.6
Widowed	11	16.9
Type of housing		
Leased	11	16.9
Assigned	3	4.6
Own	51	78.5
Education		
Little education	45	69.2
Average education	16	24.6
Upper level	4	6.2
Physical activity		
No	58	89.2
Yes	7	10.8
**Economic profile**		
Companion works out		
No	43	66.2
Yes	19	29.2
NI	3	4.6
Paid activity		
No	55	84.6
Yes	10	15.4
Signed wallet		
No	59	90.8
Yes	6	9.2
Salary		
No salary	7	10.8
1 to 2	33	50.8
3 to 4	25	38.5

NI = not informed.

**Table 2:** Characterization of the social profile (*n* = 65).

Social Profile	*n*	%
How many people in the house		
A person	5	7.7
2 to 3 people	40	61.5
>3 people	20	30.8
N° Children		
Do not have children	10	15.4
A son	11	16.9
2 to 3 children	27	41.5
>3	17	26.2
Lives with someone		
No	24	36.9
Yes	37	56.9
NI	4	6.2
Religion		
Catholic	37	56.9
Spiritist	5	7.7
Evangelical	18	27.7
NI	5	7.7
Active in religion		
No	14	21.5
Yes	50	76.9
NI	1	1.5
Social benefit		
No	7	10.8
Yes	58	89.2
Has caregiver		
No	32	49.2
Yes	33	50.8
Family support for treatment		
No	3	4.6
Yes	62	95.4

NI = not informed.

**Table 3:** Prevalence and Poisson regression analysis of the association of pain sites with severe pain interference in activities of daily living (*n* = 65).

Variables	Head			Trunk			Upper Limb			Lower Limb		
	*n* (%)	PR (95% CI)	*p*	*n* (%)	PR (95% CI)	*p*	*n* (%)	PR (95% CI)	*p*	*n* (%)	PR (95% CI)	*p*
*Pain interference*												
In its general activity			0.754 *			0.664			0.129			0.118
No	4 (33.3%)	1		13 (36.1%)	1		6 (26.1%)	1		12 (30.8%)	1	
Yes	8 (66.7%)	1.25 (0.42–3.75)		23 (63.9%)	1.11 (0.69–1.76)		17 (73.9%)	1.77 (0.80–3.90)		27 (69.2%)	1.41 (0.88–2.23)	
At your disposal			0.524 *			0.594			0.089			0.476
No	4 (33.3%)	1		15 (41.7%)	1		7 (30.4%)	1		16 (41.0%)	1	
Yes	8 (66.7%)	1.61 (0.53–4.86)		21 (58.3%)	1.13 (0.72–1.77)		16 (69.6%)	1.84 (0.87–3.88)		23 (59%)	1.16 (0.77–1.75)	
In his ability to walk			0.531 *			0.447			0.003 *			0.152
No	4 (33.3%)	1		14 (38.9%)	1		4 (17.4%)	1		14 (35.9%)	1	
Yes	8 (66.7%)	1.51 (0.50–4.56)		22 (61.1%)	1.19 (0.75–1.88)		19 (82.6%)	3.59 (1.37–9.46)		25 (64.1%)	1.35 (0.87–2.09)	
In your normal work (includes both domestic work and work outside the home)			0.215 *			0.718			0.085			0.265
No	8 (66.7%)	1		17 (47.2%)	1		8 (34.8%)	1		17 (43.6%)	1	
Yes	4 (33.3%)	0.48 (0.16–1.46)		19 (52.8%)	1.08 (0.70–1.68)		15 (65.2%)	1.82 (0.89–3.70)		22 (56.4%)	1.25 (0.83–1.89)	
In your relationships with other people			0.213			0.629			0.814			0.150
No	5 (41.7%)	1		22 (61.1%)	1		13 (56.5%)	1		20 (51.3 %)	1	
Yes	7 (58.3%)	1.97 (0.69–5.60)		14 (38.9%)	0.89 (0.57–1.41)		10 (43.5%)	1.08 (0.56–2.11)		19 (48.7%)	1.34 (0.90–1.98)	
In your sleep			0.114 *			0.975			0.700			0.415
No	4 (33.3%)	1		20 (55.6%)	1		12 (52.2%)	1		20 (51.3%)	1	
Yes	8 (66.7%)	2.48 (0.82–7.49)		16 (44.4%)	0.99 (0.64–1.55)		11 (47.3%)	1.14 (0.59–2.20)		19 (48.7%)	1.18 (0.79–1.75)	
In your pleasure of living			0.717 *			0.814			0.769			0.392
No	8 (66.7%)	1		27 (75%)	1		18 (78.3%)	1		27 (69.2%)	1	
Yes	4 (33.3%)	1.41 (0.48–4.13)		9 (25%)	0.94 (0.56–1.58)		5 (21.7%)	0.78 (0.34–1.79)		12 (30.8%)	1.25 (0.84–1.87)	

CI: confidence interval; PR: adjusted prevalence ratio. Fisher’s exact test * was used for frequencies below five. *p* < 0.05 was considered statistically significant (bold mark).

## Data Availability

The data presented in this study are available on request from the corresponding author.
